# Fibrotic microenvironment promotes the metastatic seeding of tumor cells via activating the fibronectin 1/secreted phosphoprotein 1-integrin signaling

**DOI:** 10.18632/oncotarget.10157

**Published:** 2016-06-18

**Authors:** Chong Zhang, Mengzhi Wu, Lizhen Zhang, Li-Ru Shang, Jian-Hong Fang, Shi-Mei Zhuang

**Affiliations:** ^1^ Key Laboratory of Gene Engineering of the Ministry of Education, State Key Laboratory of Biocontrol, Collaborative Innovation Center for Cell Signaling Network, School of Life Sciences, Sun Yat-Sen University, Guangzhou, P.R. China

**Keywords:** fibrotic microenvironment, metastatic seeding, FN1, SPP1, ITGAV

## Abstract

The seeding of tumor cells is a critical step in the process of metastasis, but whether and how the microenvironment of target organs affects metastatic seeding remain largely unknown. Based on cell and mouse models, we found that the metastatic seeding and outgrowth of tumor cells were significantly enhanced in fibrotic lungs. The conditioned medium from both fibrotic lungs and the fibrotic lung-derived fibroblasts (CM-FLF) had a strong activity to chemoattract tumor cells and to inhibit the apoptosis of tumor cells. Subsequent investigations revealed that the levels of fibronectin 1 (FN1) and secreted phosphoprotein 1 (SPP1) were significantly increased in fibrotic lungs. Silencing of FN1 in the fibrotic lung-derived fibroblasts dramatically decreased the chemoattracting activity of CM-FLF, while silencing of FN1 or SPP1 in fibroblasts attenuated the anti-apoptosis activity of CM-FLF. Moreover, the CM-FLF-induced apoptosis resistance or chemotaxis of tumor cells was attenuated when ITGAV, the common receptor of FN1 and SPP1, was silenced by RNA interference or blocked by GRGDS treatment in tumor cells. Consistently, ITGAV silencing or GRGDS treatment significantly inhibited the seeding and outgrowth of tumor cells in fibrotic lungs *in vivo*. Collectively, we suggest that fibrotic microenvironment may enhance the metastatic seeding of tumor cells in the lung by chemoattracting tumor cells and inhibiting their apoptosis via activating the FN1/SPP1-ITGAV signaling. These findings give a novel insight into the regulatory mechanisms of cancer metastasis and provide a potential target for anti-metastasis therapy.

## INTRODUCTION

Cancer metastasis is a complex, multi-step process that tumor cells detach from primary site, migrate and invade through extracellular matrix, intravasate into vessels, survive in the bloodstream, extravasate from vessels, seed in target organ and grow to form micro- and macro-metastatic nodules [1, 2]. Previous studies have focused on exploring the mechanisms that confer tumor cells with the ability to detach, migrate, invade, and resist anoikis. To date, whether and how the microenvironment of target organs affects the seeding and outgrowth of tumor cells in the metastatic sites remain largely unknown.

Lung is one of the most common metastatic sites for various types of cancer cells, including hepatocellular carcinoma and breast cancer [3–5]. Pulmonary fibrosis is characterized by abnormal alveolar structure, myofibroblast accumulation and collagen deposition [6, 7]. Recently, three studies based on animal models indicate that pulmonary fibrosis may promote the survival and outgrowth of metastatic mammary and lung carcinoma cells in the lungs [8–10]. Mechanically, TGF-β may induce an increased proportion of regulatory T cells and a decreased fraction of activated effector T cells in fibrotic lungs, thus creating an immunosuppressive environment [8]. Barkan *et al.* show that the type-I collagen-enriched fibrotic environment in the lung induces the metastatic growth of dormant mammary cancer cell through activation of SRC and focal adhesion kinase [9]. Experimental evidences also reveal that collagen crosslinking may create a growth-permissive fibrotic microenvironment that supports metastatic growth by enhancing tumor cell survival [10]. These emerging evidences suggest the significance of fibrotic microenvironment on the outgrowth of tumor cells in the lungs. Obviously, more extensive investigations are required to explore the impact of fibrotic microenvironment on the seeding of tumor cells and to identify the molecules that mediate the pro-metastasis effect of fibrotic microenvironment.

Based on *in vitro* and *in vivo* experiments, we explored the impact of fibrotic microenvironment on the seeding of tumor cells. The results disclosed that fibrotic microenvironment enhanced the seeding of tumor cells and thereby the metastatic growth of tumor cells in the lung. Furthermore, fibronectin 1 (FN1) and secreted phosphoprotein 1 (SPP1) secreted by the fibrotic lung-derived fibroblasts promoted the chemotaxis and the apoptosis resistance of tumor cells via FN1/SPP1-Integrin αv (ITGAV) signaling, thereby facilitating the seeding and outgrowth of tumor cells in the lung. These results provide a novel insight into the role of FN1 and SPP1 in the metastatic seeding of tumor cells and implicate the FN1/SPP1-ITGAV signaling as a potential therapeutic target for metastasis.

## RESULTS

### Fibrotic microenvironment promotes the metastatic seeding and outgrowth of tumor cells in the lungs

**Figure 1:**
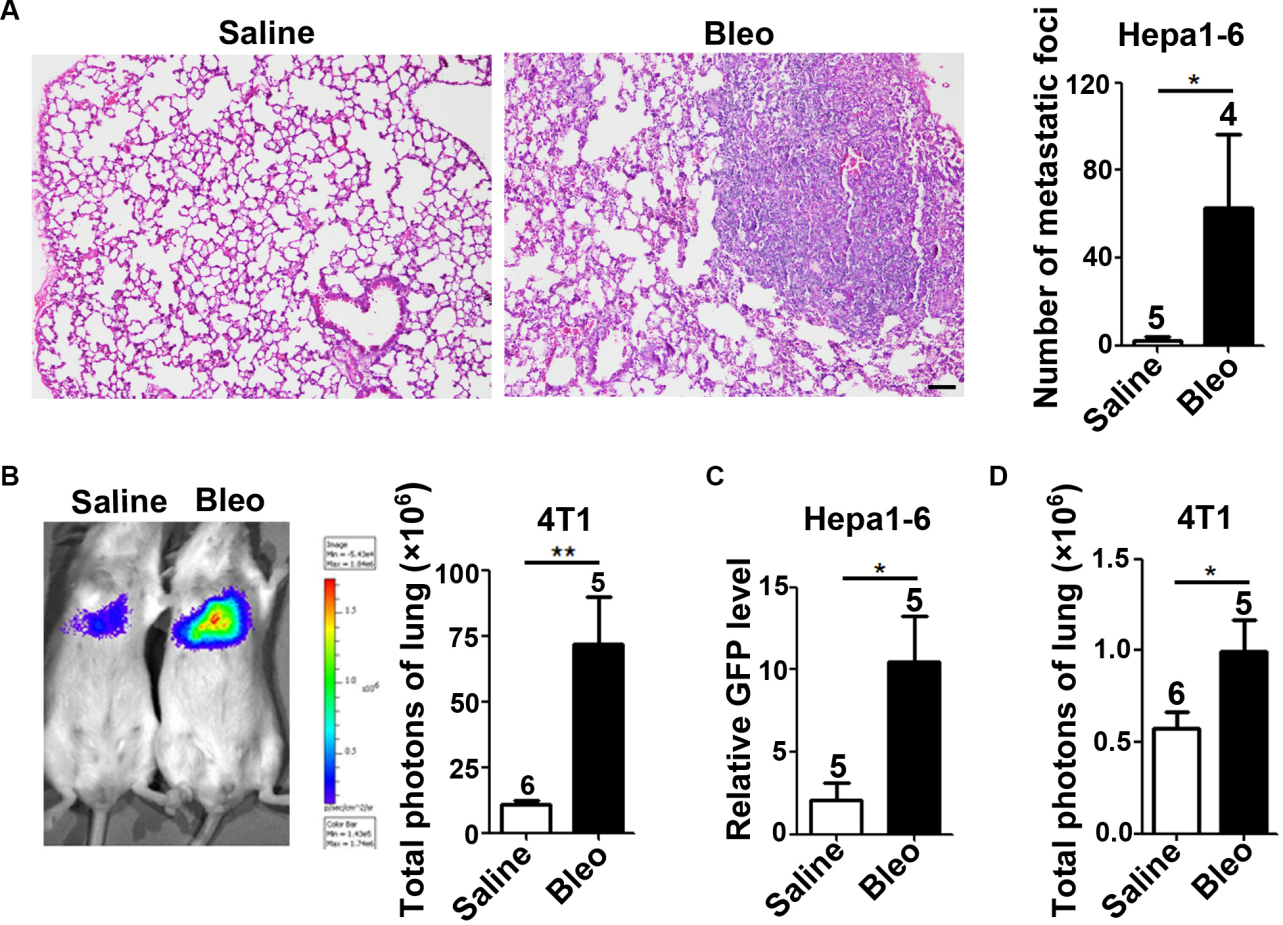
Fibrotic microenvironment promotes the seeding and outgrowth of tumor cells in the lungs (**A**, **B**) Fibrotic microenvironment promoted the outgrowth of tumor cells in the lungs. Hepa1-6-GFP (A, 2 × 10^6^) and 4T1-luc (B, 2 × 10^5^) cells were injected into the tail vein of saline or bleomycin-treated C57BL/6 and BALB/c mice, respectively. Metastasis burden was analyzed by HE staining 21 days post-injection (A, Hepa1-6-GFP) or monitored by *in vivo* bioluminescence imaging nine days after inoculation (B, 4T1-luc). Scale bar, 100 μm in (A). (**C**, **D**) Fibrotic microenvironment enhanced the seeding of tumor cells in the lungs. Hepa1-6-GFP (C, 1 × 10^6^) and 4T1-luc (D, 1 × 10^6^) cells were injected into the tail vein of saline or bleomycin-treated C57BL/6 and BALB/c mice, respectively. Ten hours later, murine lungs were subjected to the realtime quantitative PCR (qPCR) for the mRNA levels of GFP (C) or the *in vivo* bioluminescence imaging (D). The number of mice in each group is indicated on the top of cartogram. *P < 0.05; **P < 0.01.

To evaluate whether and how the fibrotic environment affects the metastatic seeding of tumor cells, we first established a pulmonary fibrosis model by intratracheal instillation of bleomycin (Supplementary Figure S1). Mouse hepatoma cell line Hepa1-6-GFP and mammary tumor cell line 4T1-luc that stably expressed GFP and luciferase, respectively, were injected into the tail vein of saline or bleomycin-treated mice. Three weeks later, pulmonary metastatic burden of Hepa1-6-GFP cells was examined by hematoxylin-eosin (HE) staining and the metastatic foci was confirmed by immunohistochemical staining of GFP (Supplementary Figure S2). Compared with saline-treated group, the frequency of pulmonary metastasis (saline vs bleomycin groups: 1/5 vs 4/4) and the number of metastatic foci (Figure 1A) were significantly increased in the bleomycin-treated mice. Moreover, *in vivo* bioluminescence imaging was employed to determine the metastasis of 4T1-luc cells nine days after injection. Consistent with the findings from Hepa1-6-GFP cells, luciferase signal in the lungs of bleomycin-treated mice was six times stronger than that of saline-treated mice (Figure 1B). These results indicate that fibrotic microenvironment may promote the metastasis of tumor cells to the lung.

Seeding of tumor cells in the target organ is a critical step in metastasis process, we therefore evaluated whether fibrotic microenvironment affected the seeding of tumor cells. Ten hours after intravenous injection of Hepa1-6-GFP cells, the mRNA level of GFP in the lungs, which represented the amount of seeding tumor cells, was analyzed by quantitative PCR (qPCR). GFP level in the lungs of bleomycin-treated mice was much higher than that of saline-treated mice (Figure 1C). Consistently, ten hours after intravenous injection of 4T1-luc cells, luciferase signal, which represented the number of 4T1-luc cells, significantly increased in the lungs of bleomycin-treated mice compared to that of saline-treated mice (Figure 1D). These results imply that fibrotic microenvironment may significantly increase the metastasis of tumor cells by enhancing the seeding of tumor cells.

### Fibrotic microenvironment chemoattracts tumor cells and enhances the resistance of tumor cells to apoptosis

Chemotaxis is important for tumor cells to extravasate from vessels and seed in the target organ [11, 12]; while apoptosis determines the amount of living tumor cells that seed and consequently outgrow in the target organ [2]. We therefore investigated whether fibrotic microenvironment affected the seeding by regulating the chemotaxis and apoptosis of tumor cells.

**Figure 2:**
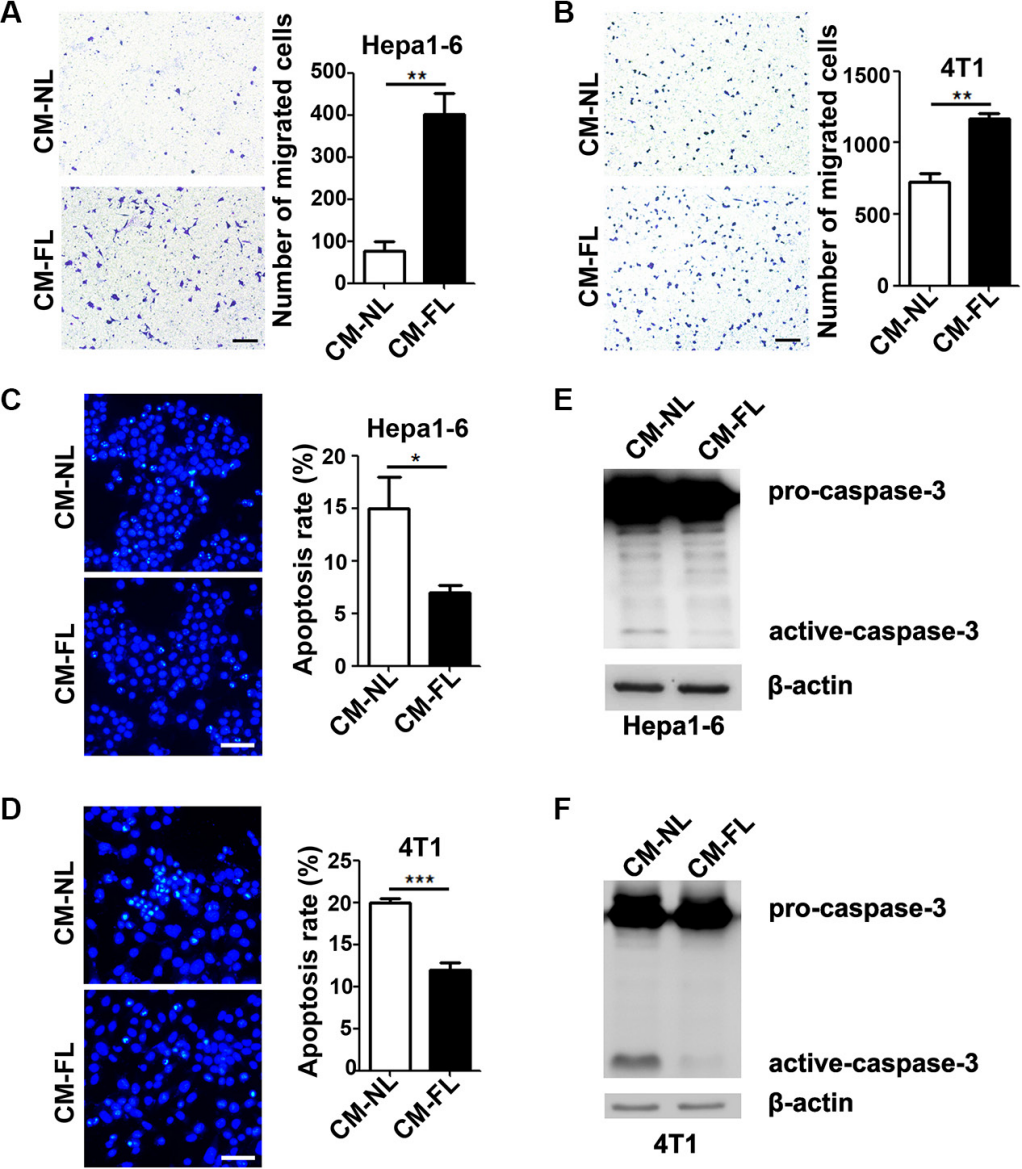
The conditioned medium from fibrotic lungs has chemoattracting and anti-apoptosis activity (**A**, **B**) The conditioned medium from fibrotic lungs enhanced the chemotaxis of tumor cells. Serum-free CM-NL or CM-FL was added to the lower chamber of transwell, while Hepa1-6 (A) and 4T1 (B) cells were added to the upper chamber. Total number of migrated cells from five random fields (100×) was calculated for each sample. Scale bar, 100 μm. (**C**, **D**) CM-FL inhibited the apoptosis of tumor cells. Hepa1-6 (C) and 4T1 (D) cells were cultured with serum-free CM-NL or CM-FL for 48 hours. Apoptosis was analyzed by 4′-6′-diamidino-2-phenylindole (DAPI) staining and at least 500 cells were counted for each sample. Scale bar, 50 μm. (**E**, **F**) CM-FL decreased the level of active-caspase-3 in tumor cells. Hepa1-6 (E) and 4T1 (F) were cultured in serum-free CM-NL or CM-FL for 22 hours, followed by detection for pro- and active-caspase-3 using immunoblotting. β-actin, internal control. Data are derived from three independent experiments. *P < 0.05; **P < 0.01; ***P < 0.001.

Transwell chamber assay was used to examine the chemoattracting activity of fibrotic lungs *in vitro*. Tumor cells were seeded in the upper chamber, while the conditioned medium from fibrotic lungs (CM-FL) or normal lungs (CM-NL) was added to the lower chamber. As shown, CM-FL displayed much more strong activity than CM-NL to chemoattract Hepa1-6 and 4T1 cells (Figure 2A and 2B). Furthermore, compared with Hepa1-6 and 4T1 cells that were cultured with serum-free CM-NL, those grown in serum-free CM-FL had significantly lower rates of apoptosis, as detected by morphological examination and fluorescence-activated cell sorter (FACS) (Figure 2C and 2D, Supplementary Figure S3). Consistently, the level of active caspase-3 was decreased in Hepa1-6 and 4T1 cells that were cultured in serum-free CM-FL (Figure 2E and 2F), indicating that fibrotic microenvironment may promote the chemotaxis of tumor cells to the lungs and inhibit the apoptosis of tumor cells seeded in the lungs.

**Figure 3:**
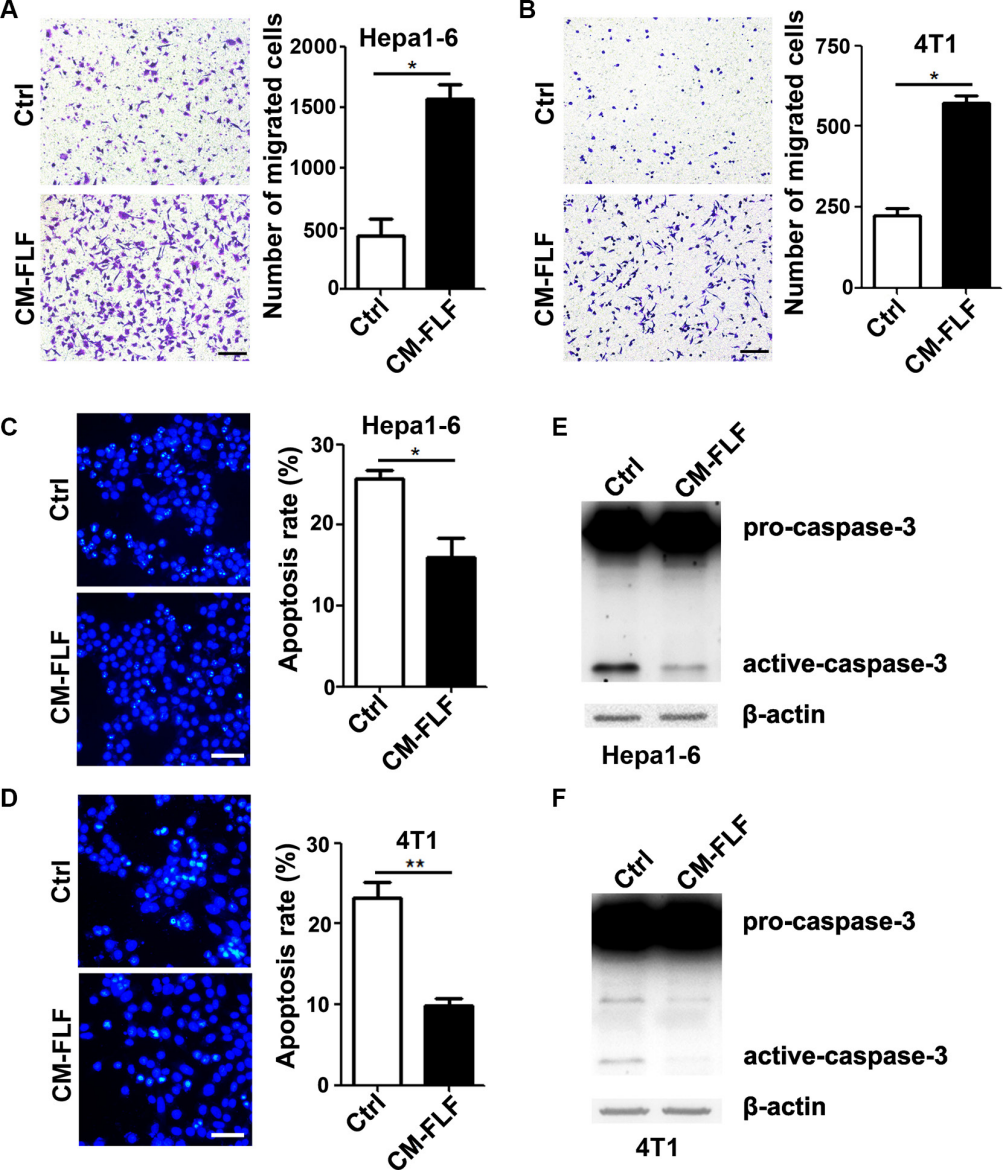
The conditioned medium of fibrotic lung-derived fibroblasts possesses chemoattracting and anti-apoptosis activity (**A**, **B**) The conditioned medium of fibrotic lung-derived fibroblasts had a strong chemoattracting activity. Ctrl (control) medium containing 1% FBS or CM-FLF containing 1% FBS was added to the lower chamber of transwell, while Hepa1-6 (A) and 4T1 (B) cells were added to the upper chamber. Scale bar, 100 μm. (**C**, **D**) CM-FLF inhibited the apoptosis of tumor cells. Hepa1-6 (C) and 4T1 (D) cells were grown in serum-free Ctrl medium or CM-FLF for 36 hours, followed by DAPI staining. Scale bar, 50 μm. (**E**, **F**) CM-FLF decreased the level of active-caspase-3 in tumor cells. Hepa1-6 (E) and 4T1 (F) were cultured in serum-free Ctrl medium or CM-FLF for 22 hours, followed by detection for pro- and active-caspase-3 using immunoblotting. β-actin, internal control. Data are derived from three independent experiments. *P < 0.05; **P < 0.01.

Fibroblast is the primary responding cell that is activated during fibrosis [6, 13]. Consistent with previous studies, an obvious increase in the number of fibroblasts was observed in fibrotic lungs (Supplementary Figure S4). We therefore isolated fibroblasts from the lungs of bleomycin-treated mice and evaluated whether the conditioned medium from the fibrotic lung-derived fibroblasts (CM-FLF) could mimic the function of CM-FL. Compared with the group of control medium, much more Hepa1-6 and 4T1 cells migrated towards CM-FLF (Figure 3A and 3B). Moreover, the apoptosis rate (Figure 3C and 3D) and the level of active caspase-3 (Figure 3E and 3F) were significantly decreased in Hepa1-6 and 4T1 cells that were grown in serum-free CM-FLF, compared with those in control medium. These results suggest that the activated fibroblasts may contribute to the fibrotic lung-promoted apoptosis resistance and chemotaxis of tumor cells.

### Fibrotic microenvironment promotes the chemotaxis and the apoptosis resistance of tumor cells via FN1/SPP1-ITGAV signaling

As shown above, the conditioned medium from both fibrotic lungs and fibrotic lung-derived fibroblasts had chemoattracting and anti-apoptosis roles on tumor cells, suggesting that secreted molecules play an essential role in these processes. We therefore screened for the fibroblast-secreted proteins that had both chemoattracting and apoptosis-inhibitory functions. FN1, SPP1, CXCL12 and vitronectin (VTN) are known to promote the survival and migration of tumor cells [14–17]. We found that mRNA levels of *Fn1* and *Spp1* were significantly increased in the fibrotic lungs of both C57BL/6 and BALB/c mice, whereas *Cxcl12* was only up-regulated in C57BL/6 but not BALB/c mice and *Vtn* was down-regulated in both strains (Supplementary Figure S5A). Immunohistochemical staining confirmed the increase of FN1 and SPP1 proteins in the fibrotic lungs (Supplementary Figure S5B). We therefore focused on FN1 and SPP1 for further investigation.

**Figure 4:**
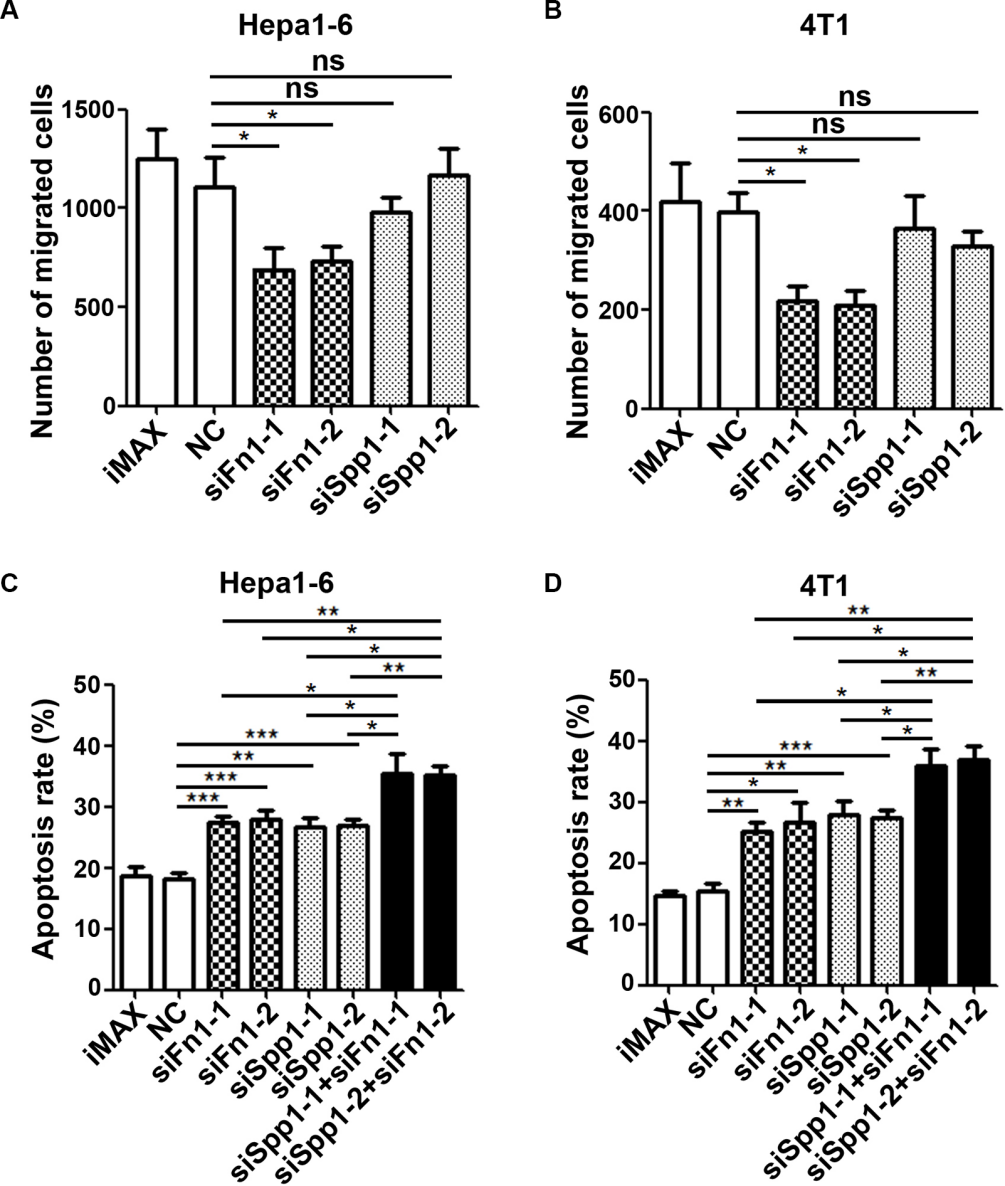
Silencing of FN1 or SPP1 in fibrotic lung-derived fibroblasts attenuates the chemoattracting and anti-apoptosis activity of CM-FLF (**A**, **B**) FN1 but not SPP1 silencing in fibrotic lung-derived fibroblasts attenuated the ability of CM-FLF to chemoattract tumor cells. The fibrotic lung-derived fibroblasts were transfected with the indicated siRNAs for 48 hours, followed by refreshment with 1% FBS-supplemented RPMI 1640. The conditioned medium was harvested 24 hours later, and then applied to the lower chamber of transwell. (**C**, **D**) FN1 and SPP1 silencing in fibrotic lung-derived fibroblasts abrogated the anti-apoptosis activity of CM-FLF. The fibrotic lung-derived fibroblasts were transfected with the indicated siRNAs for 48 hours, followed by refreshment with serum-free RPMI 1640. The conditioned medium was harvested 24 hours later, and applied to incubation with Hepa1-6 (C) and 4T1 (D) for 36 hours before DAPI staining. iMAX, treatment with transfection reagent RNAiMAX. NC, transfection with negative control duplex for siRNAs. Data are derived from three independent experiments. *P < 0.05; **P < 0.01; ***P < 0.001; ns, not significant.

To explore whether FN1 and SPP1 were responsible for the chemoattracting and anti-apoptosis activity of fibroblasts, the expression of *Fn1* or *Spp1* was silenced by siRNAs in the activated fibroblasts (Supplementary Figure S6), which were isolated from the fibrotic lungs of bleomycin-treated mice. Interestingly, the conditioned medium from FN1-silencing fibroblasts displayed significantly reduced activity to chemoattract Hepa1-6 and 4T1 cells (Figure 4A and 4B, Supplementary Figure S7A and S7B), although the conditioned medium from SPP1-silencing fibroblasts had no effect. Furthermore, the serum deprivation-triggered apoptosis was increased when tumor cells were cultured in the conditioned medium from the FN1 and SPP1-silencing fibroblasts, and this effect was more prominent when both FN1 and SPP1 were silenced in fibroblasts (Figure 4C and 4D). These data indicate that FN1 and SPP1 may mediate the chemoattracting and anti-apoptosis activity of the fibrotic lung-derived fibroblasts.

**Figure 5:**
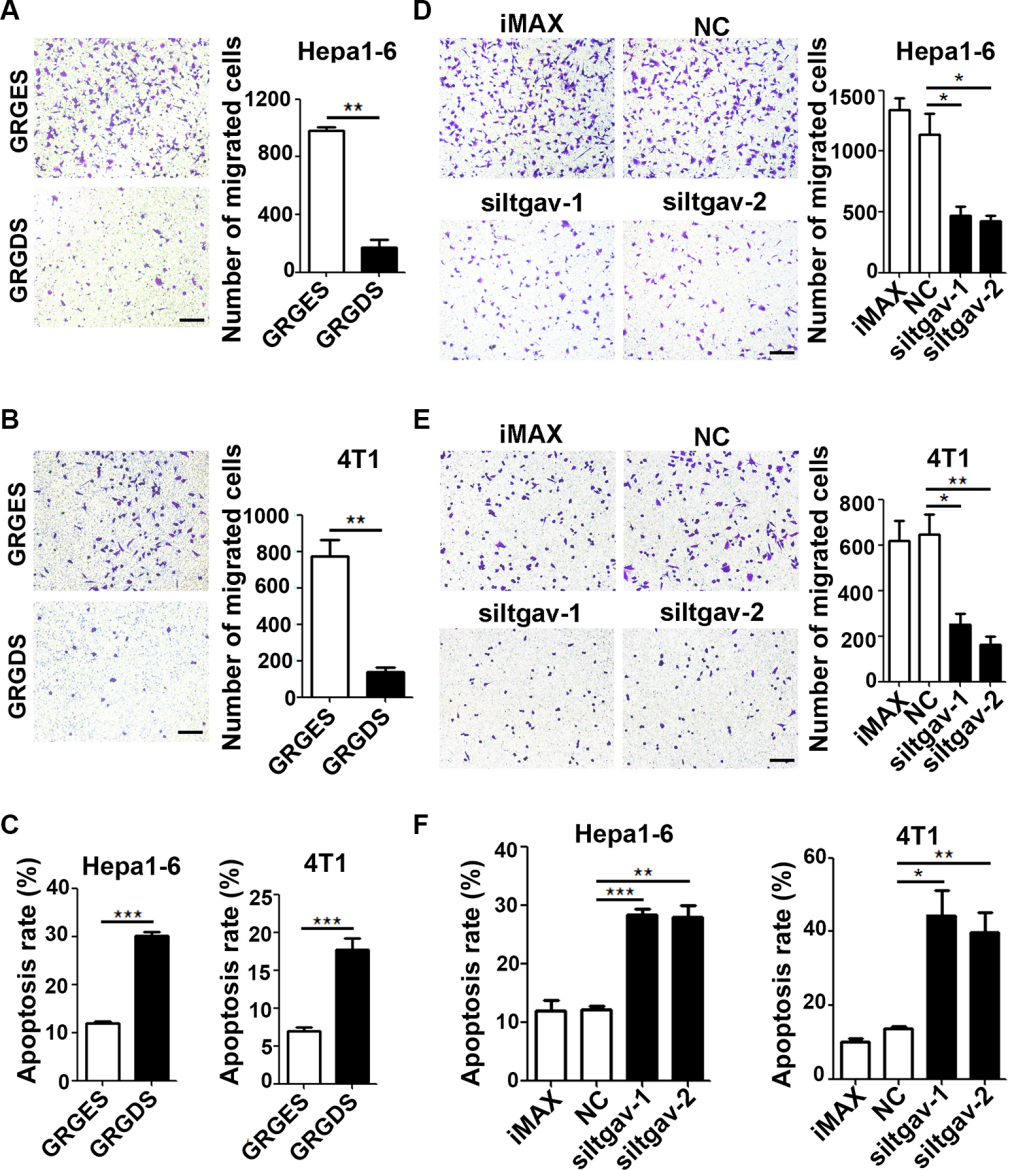
Blocking the ITGAV pathway in tumor cells attenuates the chemoattracting and anti-apoptosis activity of CM-FLF (**A**, **B**) GRGDS treatment reduced the chemotaxis of tumor cells towards CM-FLF. Hepa1-6 (A) or 4T1 (B) cells were resuspended in the medium containing 25 ug/ml GRGES (control) or GRGDS and added to the upper chamber of transwell, while CM-FLF containing 1% FBS was added to the lower chamber. (C) GRGDS treatment blocked the anti-apoptosis effect of CM-FLF on tumor cells. Hepa1-6 or 4T1 cells were cultured in the serum-free CM-FLF containing 25 ug/ml GRGES or GRGDS for 36 hours before DAPI staining. (**D**, **E**) Silencing of ITGAV decreased the chemotaxis of tumor cells towards CM-FLF. Hepa1-6 (D) or 4T1 (E) cells were transfected with the indicated siRNAs for 36 hours, then added to the upper chamber of transwell, while CM-FLF containing 1% FBS was added to the lower chamber. (**F**) Silencing of ITGAV blocked the anti-apoptosis effect of CM-FLF on tumor cells. Hepa1-6 or 4T1 cells were transfected with the indicated siRNAs for 24 hours, then replaced with serum-free CM-FLF for 36 hours before DAPI staining. iMAX, treatment with transfection reagent RNAiMAX. NC, transfection with negative control duplex for siRNAs. Scale bar, 100 μm. Data are derived from three independent experiments. *P < 0.05; **P < 0.01; ***P < 0.001.

ITGAV is the common receptor for both FN1 and SPP1. FN1 and SPP1 bind to ITGAV through a RGD-containing domain, so their interaction can be blocked by a synthetic peptide GRGDS [18, 19]. Compared with control peptide GRGES, treatment with GRGDS caused a significant decrease in the number of Hepa1-6 and 4T1 cells that migrated towards CM-FLF (Figure 5A and 5B) and an obvious increase in the apoptosis rate of tumor cells that were cultured in serum-free CM-FLF (Figure 5C). These results were reproducible when ITGAV expression in tumor cells was silenced by siRNAs (Supplementary Figure S8 and Figure 5D–5F). In agreement with these findings, treating tumor cells with GRGDS or siRNAs against ITGAV also attenuated the chemoattracting and anti-apoptosis effect of CM-FL (Supplementary Figure S9).

Collectively, we suggest that the FN1/SPP1-ITGAV pathway plays an important role in the chemotaxis of tumor cell to fibrotic lungs and in the apoptosis resistance of seeding tumor cells in the lungs.

### Inhibition of the FN1/SPP1-ITGAV signaling prevents the fibrosis-enhanced metastatic seeding and outgrowth of tumor cells *in vivo*

**Figure 6:**
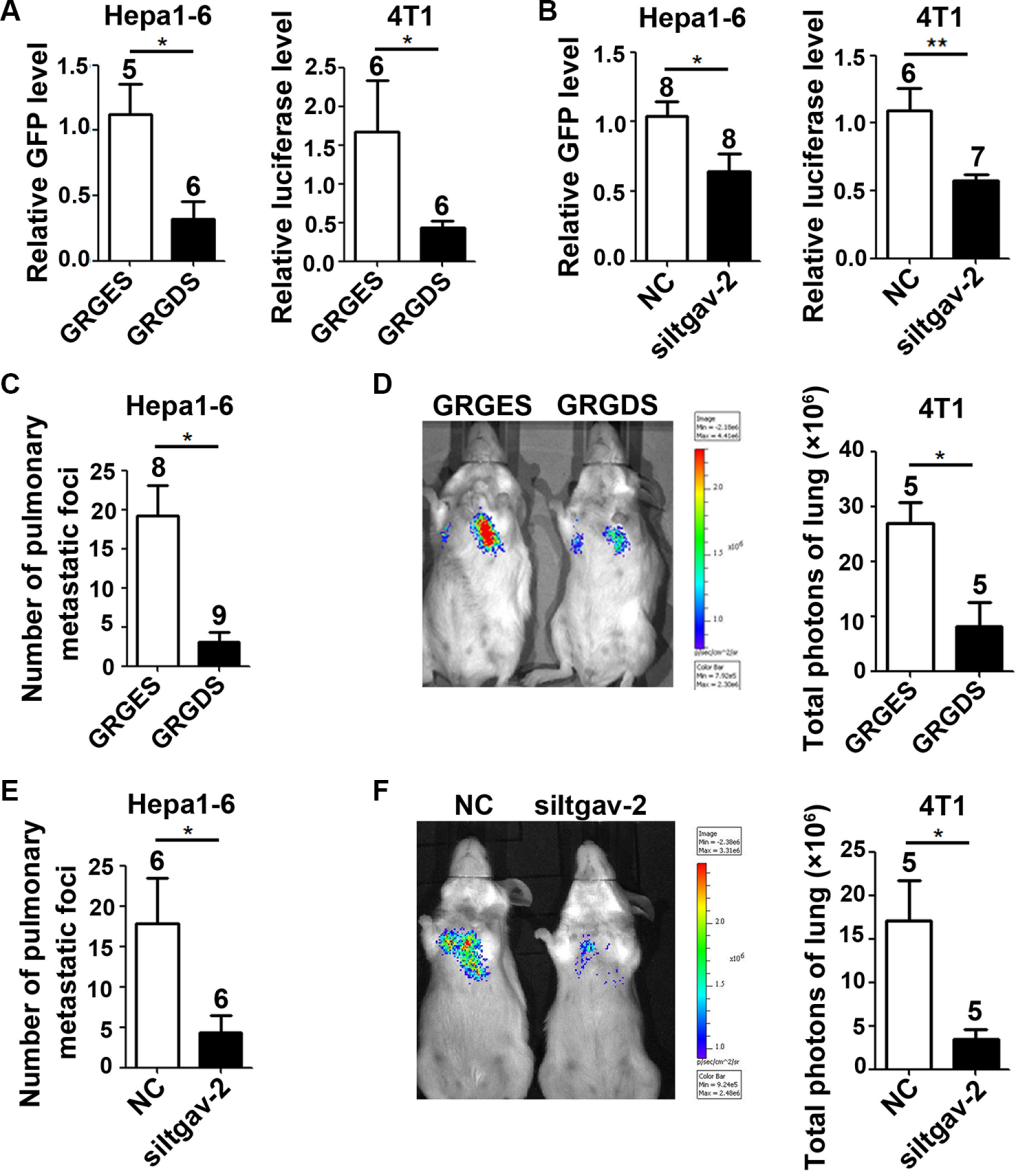
Blocking the ITGAV pathway in tumor cells inhibits the seeding and outgrowth of tumor cells in murine fibrotic lungs (**A**) GRGDS treatment inhibited the seeding of tumor cells in fibrotic lungs. Fourteen days after bleomycin treatment, GRGES or GRGDS (3 mg in 100 ul) was injected into the tail vein of bleomycin-treated mice, followed by injection of Hepa1-6-GFP (1 × 10^6^) or 4T1-luc (2 × 10^5^) cells. Ten hours after injection, the amount of seeding tumor cells in murine lungs was quantified by qPCR analysis on the mRNA level of GFP or luciferase. (**B**) Silencing of ITGAV inhibited the seeding of tumor cells in fibrotic lungs. Hepa1-6-GFP (1 × 10^6^) and 4T1-luc (2 × 10^5^) cells that were transfected with NC or siItgav-2 duplex for 36 hours were injected into the tail vein of bleomycin-treated mice. Ten hours post-injection, the amount of seeding tumor cells in murine lungs was quantified by qPCR analysis. (**C**, **D**) GRGDS treatment inhibited the outgrowth of tumor cells in fibrotic lungs. GRGES or GRGDS (3 mg in 100 ul) was injected into the tail vein of bleomycin-treated mice, followed by injection of Hepa1-6-GFP (C, 2 × 10^6^) and 4T1-luc (D, 2 × 10^5^) cells. (**E**, **F**) Silencing of ITGAV attenuated the outgrowth of tumor cells in fibrotic lungs. Hepa1-6-GFP (E, 2 × 10^6^) and 4T1-luc (F, 2 × 10^5^) cells that were transfected with NC or siItgav-2 duplex for 36 hours were injected into the tail vein of bleomycin-treated mice. For (C–F), HE staining was used to analyze the metastasis of Hepa1-6-GFP cells 21 days post-injection, and *in vivo* bioluminescence imaging was employed to monitor the outgrowth of 4T1-luc cells nine days after inoculation. The number of mice in each group is indicated on the top of cartogram. NC, transfection with negative control duplex for siRNAs. *P < 0.05; **P < 0.01.

We further evaluated whether the FN1/SPP1-ITGAV pathway was essential for the fibrosis-enhanced seeding and outgrowth of tumor cells *in vivo.* Compared with control peptide GRGES, GRGDS treatment inhibited the seeding of Hepa1-6-GFP and 4T1-luc in fibrotic lungs, as evidenced by reduced levels of GFP and luciferase in the lungs (Figure 6A). Consistently, silencing of ITGAV in Hepa1-6-GFP and 4T1-luc cells also decreased their seeding in fibrotic lungs (Figure 6B). We further examined whether blocking FN1/SPP1-ITGAV signaling could inhibit the outgrowth of tumor cells in fibrotic lungs. As shown, GRGDS treatment significantly decreased the metastatic burden of Hepa1-6-GFP and 4T1-luc cells in fibrotic lungs (Figure 6C and 6D). Similarly, the outgrowth of ITGAV-silencing tumor cells in fibrotic lungs was also attenuated (Figure 6E and 6F).

Overall, these findings imply that blockade of the FN1/SPP1-ITGAV pathway may inhibit the seeding and consequently the metastasis of tumor cells in fibrotic lungs.

## DISCUSSION

The role of organ microenvironment on the metastasis of tumor cells is still unclear. In this study, we used both cell and mouse models to demonstrate that fibrotic microenvironment enhanced the seeding and consequently the outgrowth of tumor cells in the lungs by activating the FN1/SPP1-ITGAV pathway.

FN1 is an extracellular matrix protein mainly secreted by fibroblast [10]. It promotes the adhesion, survival, migration and differentiation of cells, and thereby plays an important role in the processes such as wound healing and embryonic development [14, 20–21]. SPP1 is produced by a variety of cell types, including fibroblast, osteoblast, macrophages and endothelial cells. It plays a major role in bone remodeling, cell adhesion, migration, survival and chemotaxis [22–24]. To date, the role of FN1 and SPP1 in the metastatic microenvironment is still unknown. This study highlights the significance of FN1 and SPP1 in the metastatic microenvironment based on the following evidences: First, the seeding and outgrowth of tumor cells were increased in the bleomycin-induced fibrotic lungs. And the levels of FN1 and SPP1 were significantly enhanced in fibrotic lungs. Second, both CM-FL and CM-FLF chemoattracted tumor cells and decreased the apoptosis of tumor cells, while silencing FN1 or SPP1 in fibroblasts attenuated the CM-FLF-promoted apoptosis resistance and chemotaxis of tumor cells. Third, blocking ITGAV, the common receptor of FN1 and SPP1, on tumor cells by peptide GRGDS or siRNAs abrogated the chemoattracting and anti-apoptosis activity of fibrotic lungs or fibrotic lung-derived fibroblasts and also attenuated the *in vivo* seeding and outgrowth of tumor cells in fibrotic lungs. These findings suggest that FN1 and SPP1, which are secreted by the activated fibroblasts of fibrotic lungs, may chemoattract tumor cells and inhibit their apoptosis by activating the ITGAV pathway in tumor cells.

Chemotaxis is a critical step for the extravasation of tumor cells, thus strongly promoting the seeding of tumor cells in target organs [11]. Most published studies have demonstrated the chemoattracting activity of classical chemokines. For example, classical chemokines and their cognate receptors CXCL12-CXCR4, CCL19/CCL21-CCR7 and CCL27/CCL28-CCR10 have been reported to promote the metastasis of tumor cells to normal lungs, lymph nodes and skins, respectively [12]. Herein, we revealed that a non-classical chemokine FN1 was increased in fibrotic lungs and chemoattracted tumor cells *in vitro*, thus at least partly contributed to the enhanced seeding of tumor cells in fibrotic lungs. Interestingly, although the non-classical chemoattractant SPP1 was also increased in fibrotic lungs, SPP1 silencing did not reduce the chemoattracting activity of the fibrotic lung-derived fibroblasts. This observation may be explained by the lack of SPP1 concentration gradient between tumor cells and the activated fibroblasts in this system. In agreement with this contention, we have previously shown that the macrophage-derived OPN (namely, SPP1 in mouse) chemoattracts OPN-knockdown SK-HEP-1 cells but not parental SK-HEP-1 cells [25].

Apoptosis is a major barrier that must be circumvented for tumor cells to seed in a new organ [26–28]. It is shown that ectopic expression of anti-apoptosis proteins, such as BCL2 and XIAP, confers tumor cells with resistance to apoptosis, leading to an increase of macroscopic metastasis [29, 30]. Another report shows that VCAM-1 enhances lung metastasis of breast cancer cells by triggering AKT activation in cancer cells which protects cells from TRAIL-induced apoptosis [31]. However, whether and how the apoptosis of tumor cells is affected by fibrotic microenvironment is largely unknown. We found that the conditioned medium from both fibrotic lungs and the fibrotic lung-derived fibroblasts inhibited the apoptosis of tumor cells. Furthermore, the anti-apoptosis activity of fibrotic lungs was dependent upon the fibroblast-derived FN1 and SPP1.

Higher level of integrins is known to enhance tumor progression by promoting adhesion, survival, proliferation and invasion of tumor cells [32–34]. Here we showed that blocking ITGAV pathway significantly attenuated the *in vitro* chemoattracting and anti-apoptosis activity of fibrotic lungs and repressed the *in vivo* seeding and outgrowth of tumor cells in fibrotic lungs, suggesting the essential role of ITGAV signaling in the metastasis-promoting effect of fibrotic lungs.

Taken together, we disclosed the role of chemotaxis and apoptosis in the fibrotic microenvironment-enhanced seeding of tumor cells and identified the fibroblast-derived FN1 and SPP1 as the central mediators in these processes. These findings highlight the FN1/SPP1-ITGAV signaling as potential target for anti-metastasis therapy.

## MATERIALS AND METHODS

### Cell lines

Mouse hepatoma cell line Hepa1-6 (derived from male C57BL/6 mice) was maintained in Dulbecco's modified Eagle's medium (DMEM, Corning, NY, USA) supplemented with 10% fetal bovine serum (FBS, GIBCO, NY, USA), penicillin (100 U/mL) and streptomycin (100 U/mL). Mammary cancer cell line 4T1 (from female BALB/c mice) and 4T1-luc (4T1 cells that stably expressed luciferase) were maintained in Roswell Park Memorial Institute (RPMI) 1640 medium (Corning, NY, USA) supplemented with 10% FBS, penicillin (100 U/mL) and streptomycin (100 U/mL). Hepa-1-6 and 4T1 cells were purchased from ATCC, and 4T1-luc was from Caliper Life Sciences (MA, USA) within 6 months.

Hepa1-6 that stably expressed GFP (Hepa1-6-GFP) was established using retroviral expression system. Briefly, retroviruses were generated by transiently co-transfecting HEK293T cells with the retroviral expression vector (pBABE-puro-EGFP) and packaging plasmid (pCL-Ampho, Imgenex, CA, USA) using calcium phosphate precipitation. Thirty-six hours after transfection, cells were refreshed with the complete growth medium and incubated for another 24 hours. The lentiviral supernatants were then harvested and cellular debris was removed by centrifugation at 500 g for 10 minutes. Hepa1-6 cells were then infected with retroviruses and the GFP-expressing cells were sorted by flow cytometry (Beckman Coulter, CA, USA).

### Vector construction

To generate the pBABE-puro-EGFP, a 720 bp open reading frame of EGFP under the control of CMV was cloned into a retrovirus vector pBABE-puro (Cell Biolabs, CA, USA). The construct was verified by direct sequencing.

### Pulmonary fibrosis model

Pulmonary fibrosis was implemented in male C57BL/6 and female BALB/c mice weighing 23 to 25 grams. After anesthetized with an intraperitoneal injection of sodium pentobarbital (30 mg/kg), mice were intratracheally instilled with 75 ul saline or bleomycin (2.5 mg/Kg for C57BL/6 mice and 3 mg/Kg for BALB/c mice, Nippon Kayaku Co, Takasaki, Japan) [8–10]. Fourteen days later, mice were sacrificed and the lungs were harvested. All protocols involving animals were conducted in accordance with the Guide for the Care and Use of Laboratory Animals (NIH publications Nos. 80–23, revised 1996) and according to the Sun Yat-sen University Institutional Ethical Guidelines for animal experiments.

### Tumor metastasis model

Mice were first intratracheally instilled with saline or bleomycin. Fourteen days later, tumor cells were injected into the tail vein of C57BL/6 mice (for Hepa1-6-GFP) and BALB/c (for 4T1-luc) mice [8–10]. Pulmonary metastasis burden was examined by hematoxylin-eosin staining on the lung sections 21 days post-injection (Hepa1-6-GFP) or monitored by *in vivo* bioluminescence imaging (Xenogen IVIS kinetic, Caliper life sciences, MA, USA) nine days after injection (4T1-luc). To examine the number of tumor cells (Hepa1-6-GFP and 4T1-luc) seeding in the lungs, Hepa1-6-GFP or 4T1-luc cells were injected into the tail vein of mice. Ten hours later, the lungs were monitored by *in vivo* bioluminescence imaging or harvested and then subjected to qPCR analysis for the mRNA levels of GFP or luciferase.

### Analysis of mRNA expression by qPCR

Two μg of total RNA was subjected to DNase I digestion (Fermentas, MD, USA), followed by reverse-transcription using Moloney murine leukemia virus reverse transcriptase (Promega, WI, USA) and detection of cDNA with Power SYBR^®^ Green PCR Master Mix (Applied Biosystems, CA, USA) [36]. β-actin was used as an internal control. All reactions were performed on a LightCycler^®^ 480 (Roche Diagnostics, Mannheim, Germany) and were run in triplicate. The cycle threshold (Ct) values did not differ by more than 0.5 among the triplicates. The level of target gene was normalized to that of β-actin to allow the calculation of 2^−ΔΔCt^ value.

The primers used are listed in Supplementary Table S1.

### Immunohistochemical (IHC) staining

Formalin-fixed, paraffin-embedded tissues were cut into 5-μm sections, deparaffinized with xylene, rehydrated through graded ethanol, followed by quenching of endogenous peroxidase activity in 0.3% hydrogen peroxide, and antigen retrieval by microwave heating in 10 mM citrate buffer (pH 6.0) for GFP and SPP1 or by pressure cooker heating in EDTA buffer (pH 8.0 for FN1 or pH 9.0 for FSP1) [36]. Sections were incubated at 4°C overnight with mouse monoclonal antibody against SPP1 (1:500, ZM-0174, ZSGB-BIO, China) or rabbit polyclonal antibody against GFP (1:200, LS-C154219, LSBio, WA, USA), FSP1 (1:400, ZA-0257, ZSGB-BIO, China) and FN1 (1:200, ZA-0106, ZSGB-BIO, China), then immunostained using ChemMate DAKO EnVision Detection Kit, Peroxidase/DAB, Rabbit/Mouse (DakoCytomation, Glostrup, Denmark). Subsequently, sections were counterstained with hematoxylin and mounted in non-aqueous mounting medium. For collagen assay, lung sections were stained using Sirius Red (Sigma, MO, USA) according to the manufacturer's instructions.

To evaluate protein levels, ten representative staining fields (400×) were examined for each section. The protein levels of FN1 and SPP1 were estimated by density scanning using ImagePro Plus software (Media Cybernetics, Silver Spring, USA) [36]. To detect the number of FSP1-staining fibroblasts, ten representative fields (400×) were photographed for each section and the average number of FSP1-staining cells per field is presented.

### Collection of the conditioned medium from the lungs

Fresh lungs were weighed, sliced into small pieces at about 1 mm^3^ and allowed to adhere to the 10 cm dishes containing 0.5 ml serum-free RPMI 1640 medium for 20 min, followed by refreshment with serum-free RPMI 1640 medium according to the weight of the lung (40 μl/mg of the lung) and incubation in a humid incubator (37°C, 5% CO_2_, 20% O_2_) for 24 hours. The conditioned medium from the normal lungs (CM-NL) of saline-treated mice or the fibrotic lungs (CM-FL) of bleomycin-treated mice was then collected, centrifuged sequentially at 500 g for 5 min to remove the detached cells and at 12 000 g for 10 min to discard cell debris. Aliquots of the conditioned medium were stored at −80°C until used.

### Isolation of primary lung fibroblasts

Primary lung fibroblasts were isolated from mice as previously reported [35]. Briefly, the lungs were sliced into small pieces and allowed to adhere to 10 cm dish, followed by refreshment with RPMI 1640 medium. The cultures were maintained in a humid incubator for one week and medium was refreshed every day. Once the fibroblasts were outgrown, the remaining tissue pieces and monolayer of cells were digested. The cells were resuspended in complete cell culture medium and kept still for 5 min to allow the sedimentation of tissue debris. The cells that remained in the supernatant were collected by centrifugation at 200 g for 5 min and then reseeded into a 10 cm dish containing RPMI 1640 medium. The fibroblasts were selected and enriched due to their growth advantage. Fibroblasts at passages 3–4 were used.

### Collection of the conditioned medium from the fibrotic lung-derived fibroblasts (CM-FLF)

Conditioned medium of fibroblasts was collected as previously reported [25]. The freshly isolated fibroblasts from fibrotic lungs were seeded on a 6-well plate for 24 hours, then washed once with 1 × PBS and cultured in 2 ml RPMI 1640 medium supplemented with or without 1% FBS for 24 hours. Afterwards, the conditioned medium was centrifuged sequentially at 500 g for 5 min to remove the detached cells and at 12 000 g for 10 min to discard cell debris. The number of live cells in each well was counted to allow appropriate correction for the volume of conditioned medium used in the experiments. Aliquots of the conditioned medium were stored at −80°C until used.

For chemotaxis assay, CM-FLF was harvested with RPMI 1640 medium supplemented with 1% FBS; for apoptosis assay, CM-FLF was harvested with serum-free RPMI 1640 medium.

### *In vitro* chemotaxis assay

The chemotaxis of tumor cells was analyzed using a 24-well Boyden chamber with 8-μm pore size polycarbonate membrane (Corning, NY, USA) as described [25]. The conditioned medium was added to the lower chamber, while 1 × 10^5^ Hepa1-6 or 2 × 10^4^ 4T1 cells suspended in serum-free RPMI medium was seeded in the upper chamber. Hepa1-6 cells and 4T1 cells were allowed to migrate for 12 hours and 10 hours, respectively. The migrated cells were fixed by methanol and stained with crystal violet. Total number of migrated cells from five random fields (100×) is presented for each sample.

### Apoptosis analysis

Apoptosis of tumor cells was detected by morphological examination, fluorescence-activated cell sorter (FACS) analysis and caspase activation. For morphological examination, cells were stained with 4′-6′-diamidino-2-phenylindole (DAPI) and those with condensed or fragmented nuclei were considered as apoptotic cells [37]. At least 500 cells were counted for each sample. Annexin V/PI apoptosis assay by FACS was conducted using Annexin V-FITC Apoptosis Detection Kit (cat. B32117, Biotool, TX, USA), according to the manufacturer's protocol.

The active caspase-3 was detected by immunoblotting. Proteins were separated in a polyacrylamide gel and transferred to a methanol-activated PVDF membrane (Millipore, MA, USA). The membrane was blocked in Tris-buffered saline-Tween-20 (TBST) containing 2% bovine serum albumin, and then immunoblotted subsequently with the primary and secondary antibodies. The protein level was detected using Clarity Western ECL Substrate (Bio-Rad, CA, USA). The antibodies used for immunoblotting included: mouse monoclonal antibody against β-actin (cat. BM0627, Boster, Wuhan, China), rabbit polyclonal antibody against caspase-3 (cat. 9662s, CST, MA, USA), horse anti-mouse (CST) or goat anti-rabbit (CST) HRP-conjugated secondary antibody.

### SiRNA and cell transfection

Small interfering RNAs (siRNA) were purchased from GenePharma (Shanghai, China). The small interfering RNAs siItgav, siFn1 and siSpp1 targeted the mRNAs that coded for mouse *Itgav* (NM_008402.3), *Fn1* (NM_001276408.1) and *Spp1* (NM_001204201.1), respectively. The negative control RNA duplex (NC) for siRNAs was non-homologous to any human genome sequences. Sequences of siRNAs are list in Supplementary Table S1.

Reverse transfection of siRNAs was performed using Lipofectamine-RNAiMAX (Life Technologies). A total of 50 nM RNA duplex was used for each transfection.

### Statistical analysis

Data are expressed as the mean ± standard error of the mean (SEM) from at least three independent experiments. Unless otherwise noted, the differences between two groups were analyzed by unpaired Student *t* test. Analyses were performed with GraphPad Prism (Version 4.0, GraphPad Software, Inc., San Diego, CA, USA). All statistical tests were two-sided and *P* < 0.05 was considered statistically significant.

## SUPPLEMENTARY MATERIALS FIGURES AND TABLE


