# Supplementary material for: Fibrotic microenvironment promotes the metastatic seeding of tumor cells via activating the fibronectin 1/secreted phosphoprotein 1-integrin signaling
Source: Oncotarget. 2016 Jun 18;7(29):45702–14. doi: 10.18632/oncotarget.10157 (PMC5216754; doi:10.18632/oncotarget.10157)
Supplement: Supplementary file 1 [file oncotarget-07-45702-s001.pdf]

# Fibrotic microenvironment promotes the metastatic seeding of tumor cells via activating the fibronectin 1/secreted phosphoprotein 1-integrin signaling

## Supplementary Materials

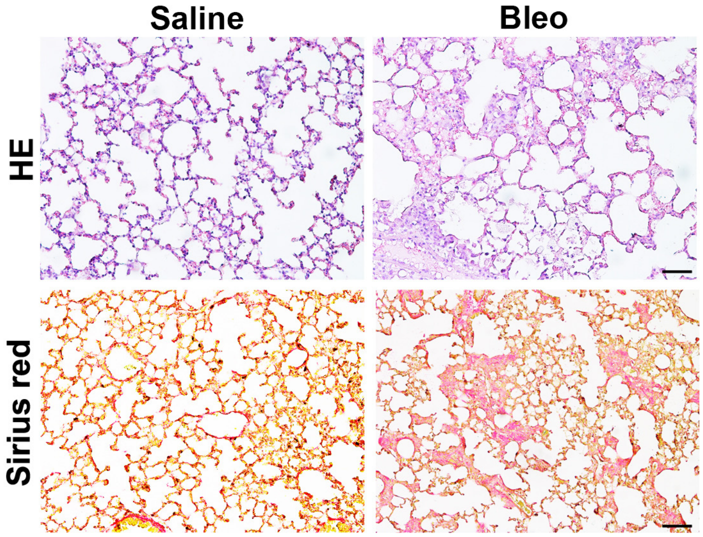


**Supplementary Figure S1: Induction of pulmonary fibrosis in C57BL/6 mice.** Fourteen days after the intratracheal instillation of saline or bleomycin (Bleo), murine lungs were harvested and subjected to hematoxylin-eosin (HE) and Sirius red staining. Scale bar, 100 μm.

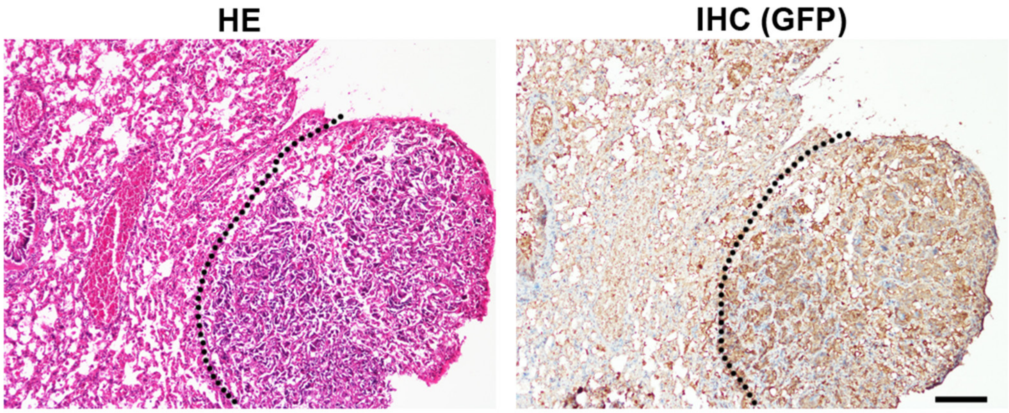


**Supplementary Figure S2: Examination of metastatic foci in murine fibrotic lung by hematoxylin-eosin (HE) staining and immunohistochemical staining with GFP.** The sections of metastatic foci were stained by HE (left) and immunohistochemically stained with GFP antibody (right). The metastatic foci were indicated by dotted lines. Scale bar, 100 μm.

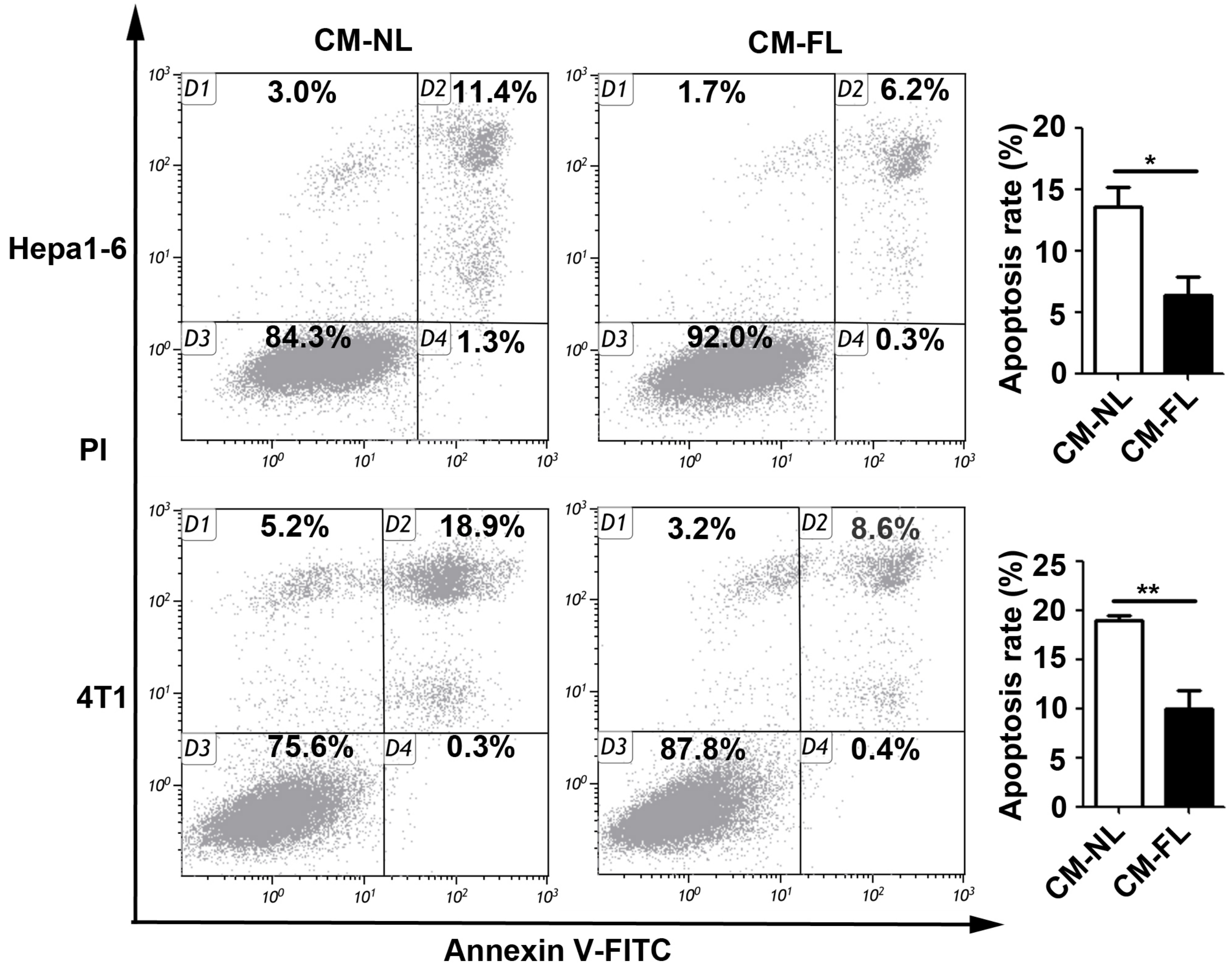


**Supplementary Figure S3: The conditioned medium from fibrotic lungs inhibited apoptosis of tumor cells.** Hepa1-6 and 4T1 cells were cultured with serum-free CM-NL or CM-FL for 48 hours. Apoptosis was analyzed by Annexin V/PI double-staining. Apoptotic cells are those stained by Annexin V but not PI (early apoptotic cells) or by both Annexin V and PI (late apoptotic cells). Data are derived from three independent experiments. *$P < 0.05$; **$P < 0.01$.

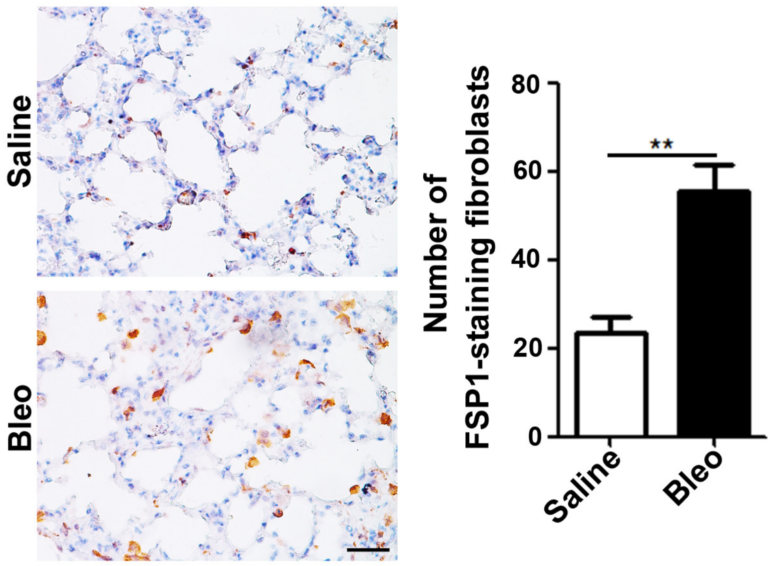


**Supplementary Figure S4: The number of fibroblasts increased in fibrotic lungs.** Fourteen days after intratracheal instillation of saline or bleomycin (Bleo) in C57BL/6 mice, the sections of lung tissues were immunohistochemically stained for FSP1. Ten random fields (400×) were photographed for each section. The average number of FSP1-staining fibroblasts per field is presented. Scale bar, 100 µm. **$P < 0.01$.

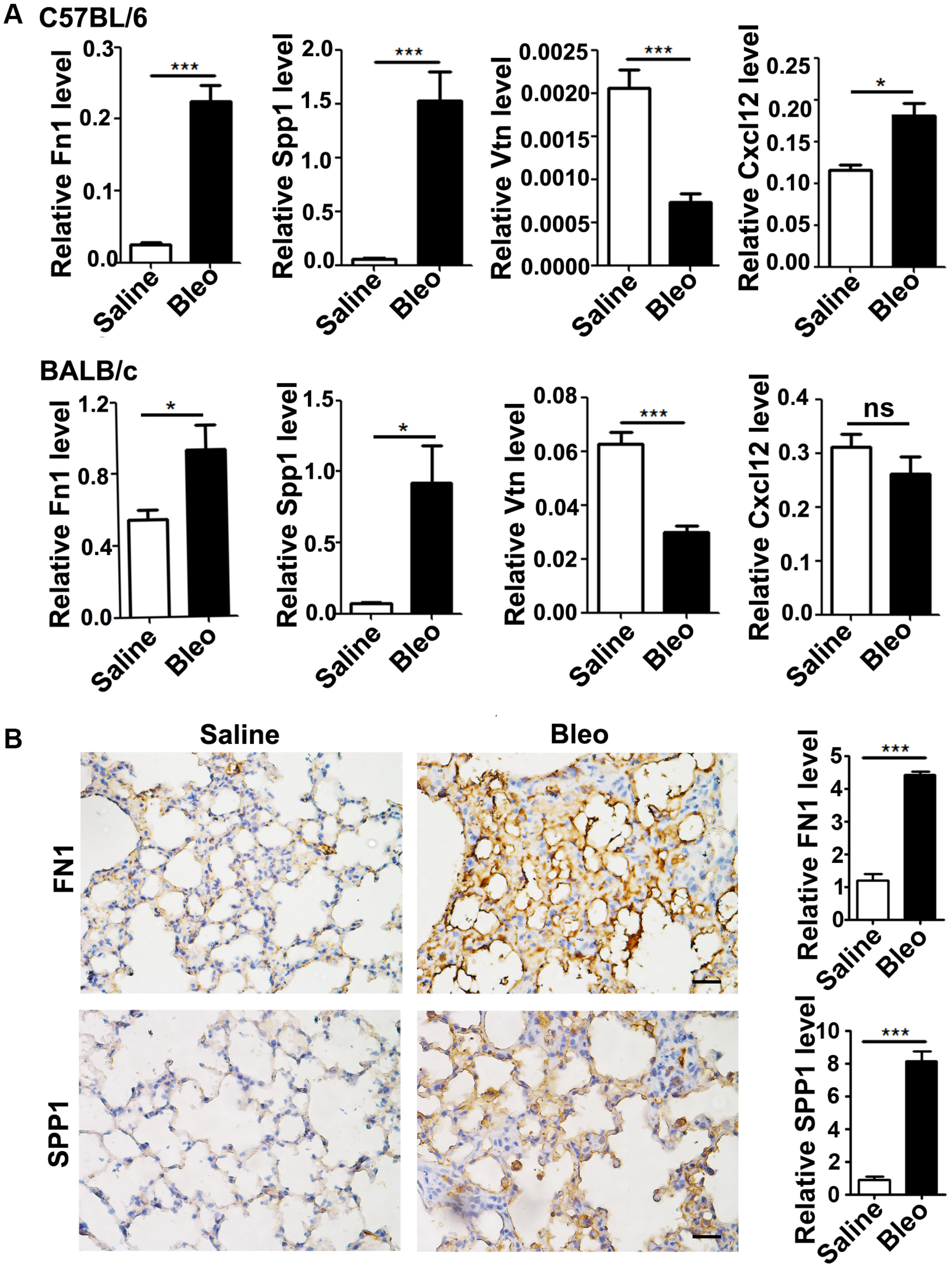


**Supplementary Figure S5: Expression of chemoattracting factors in normal and fibrotic lungs.** (**A**) The mRNA levels of chemoattracting factors. β-actin was used as a reference gene. (**B**) The levels of FN1 and SPP1 proteins were increased in fibrotic lungs. Mice were intratracheally instilled with saline or bleomycin (Bleo). Fourteen days later, the normal and fibrotic lungs of C57BL/6 and BALB/c mice were subjected to qPCR (A) and IHC (B) analysis. Scale bar, 100 μm in (B). **P* < 0.05; ****P* < 0.001; ns, not significant.

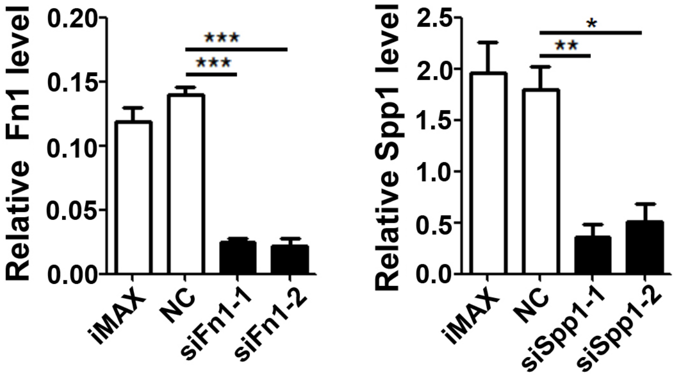


**Supplementary Figure S6: The expression of *Fn1* and *Spp1* was silenced by siRNAs.** The fibrotic lung-derived fibroblasts were transfected with the indicated duplex for 48 hours and then subjected to qPCR analysis. β-actin was used as an internal control. Data are derived from three independent experiments. **P* < 0.05; ***P* < 0.01; ****P* < 0.001.

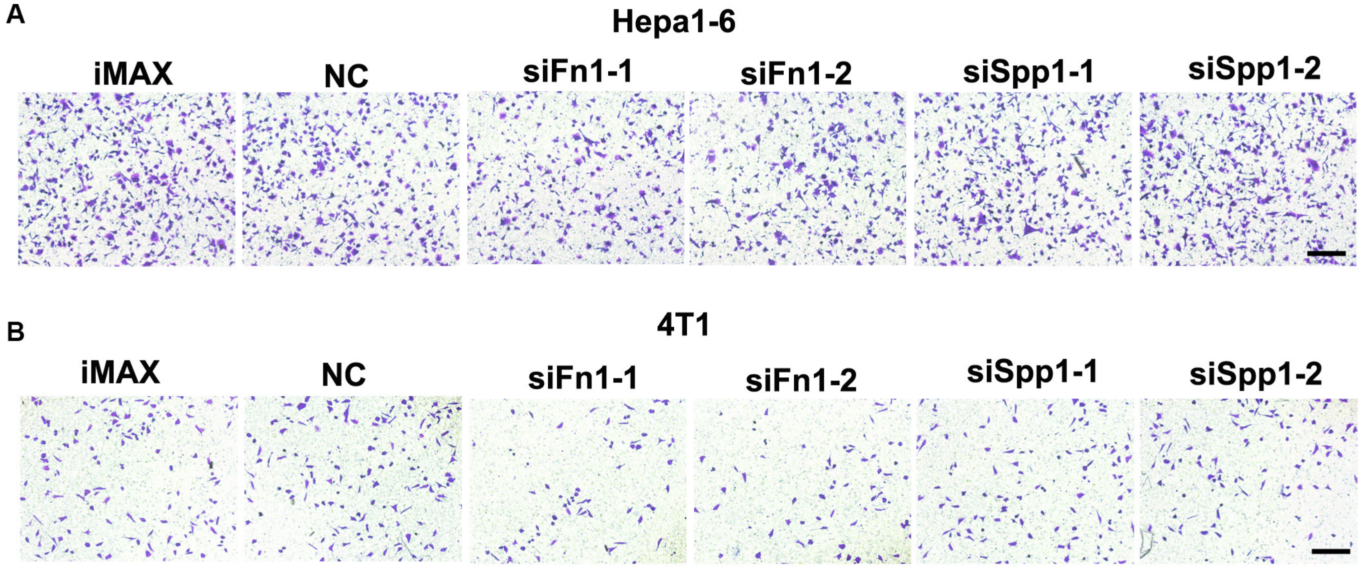


**Supplementary Figure S7: FN1 but not SPP1 silencing in the fibrotic lung-derived fibroblasts attenuated the ability of CM-FLF to chemoattract tumor cells.** Representative images of migrated cells are shown. Scale bar, 50 µm.

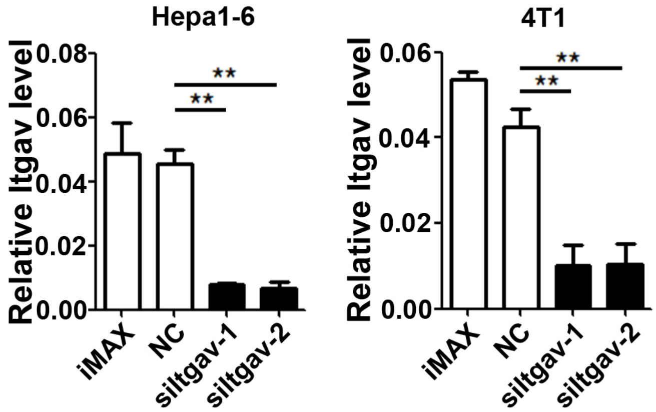


**Supplementary Figure S8: The expression of *Itgav* was silenced by siRNAs.** Hepa1-6 and 4T1 cells were transfected with the indicated duplex for 48 hours and then subjected to qPCR analysis. β-actin was used as an internal control. Data are derived from three independent experiments. ***P* < 0.01.

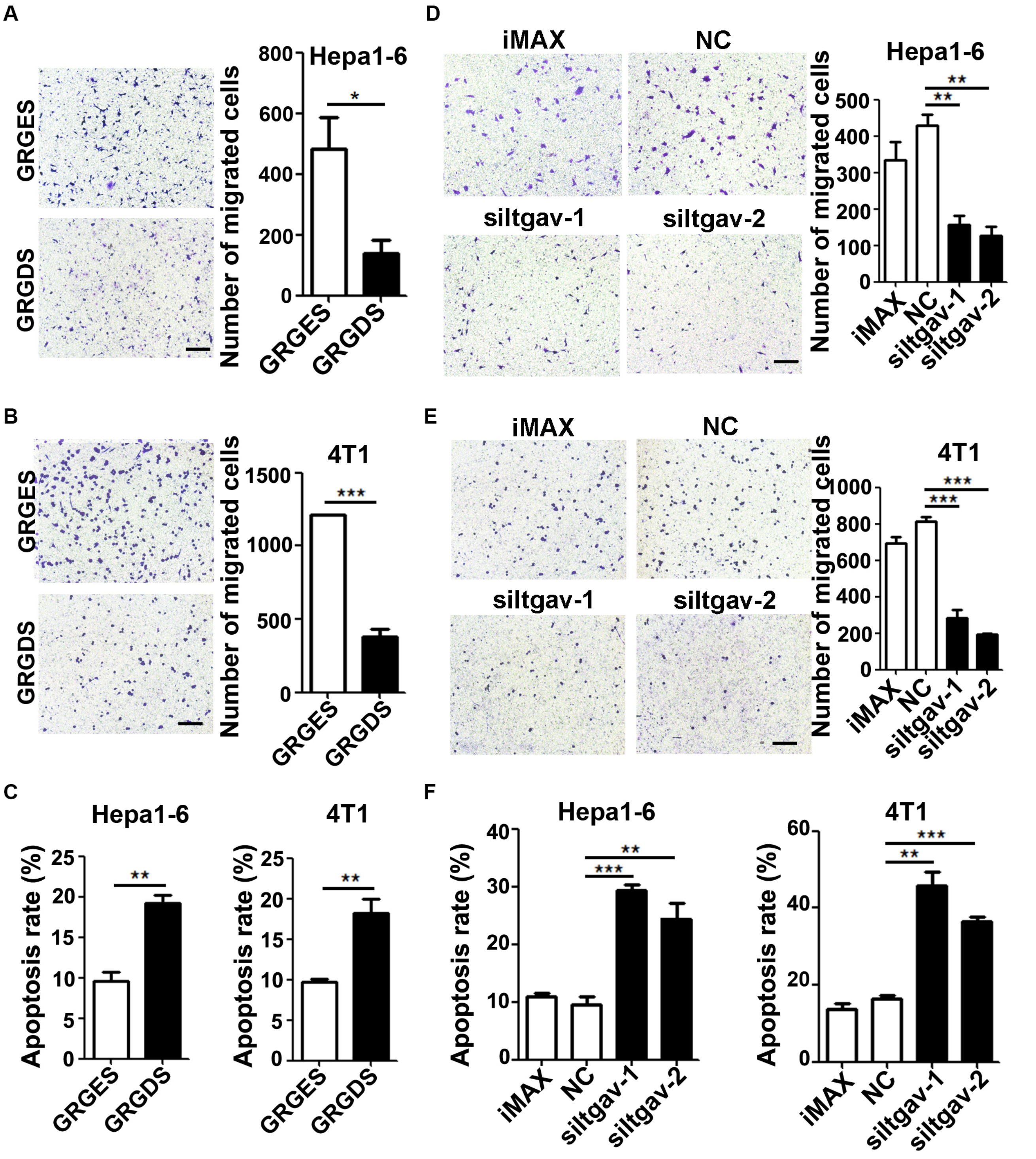


**Supplementary Figure S9: Blocking the ITGAV pathway in tumor cells attenuates the chemoattracting and anti-apoptosis effect of CM-FL.** (**A**, **B**) GRGDS treatment reduced the chemotaxis of tumor cells towards CM-FL. Hepa1-6 (A) or 4T1 (B) cells were resuspended in the medium containing 25 ug/ml GRGES (control) or GRGDS and added to the upper chamber of transwell, while CM-FL was added to the lower chamber. (**C**) GRGDS treatment blocked the anti-apoptosis effect of CM-FL on tumor cells. Hepa1-6 or 4T1 cells were cultured in the CM-FL containing 25 ug/ml GRGES or GRGDS for 48 hours before DAPI staining. (**D**, **E**) Silencing of ITGAV decreased the chemotaxis of tumor cells towards CM-FL. Hepa1-6 (D) or 4T1 (E) cells were transfected with the indicated siRNAs for 36 hours, then added to the upper chamber of transwell, while CM-FL was added to the lower chamber. (**F**) Silencing of ITGAV blocked the anti-apoptosis effect of CM-FL on tumor cells. Hepa1-6 or 4T1 cells were transfected with the indicated siRNAs for 24 hours, then replaced with CM-FL for 36 hours before DAPI staining. iMAX, treatment with transfection reagent RNAiMAX. NC, transfection with negative control duplex for siRNAs. Scale bar, 100 μm. Data are derived from three independent experiments. *$P < 0.05$; **$P < 0.01$; ***$P < 0.001$.

**Supplementary Table S1: Sequences of RNA and DNA Oligonucleotides**

| Name | Sense strand/Sense primer (5′-3′) | Antisense strand/Antisense primer (5′-3′) |
|---|---|---|
| **Primers for RT-PCR** | | |
| GFP | AGAACGGCATCAAGGTGAAC | TGCTCAGGTAGTGGTTGTCG |
| luciferase | GAGGCTAAGGTGGTGGACTT | CCACGATGAAGAAGTGCTCG |
| Fn1 | CGAGGAAACCTGCTTCAGTG | TGCTTGTTTCCTTGCGACTT |
| Spp1 | AAGCAGAATCTCCTTGCGCC | AGCAGTGACGGTCTCATCAGA |
| Vtn | TGAGCTAGATGAGACGGCAG | CATCCTCAAAGCGCCAGTAC |
| **siRNA duplexes** | | |
| siItgav-1 | CGAGGGAAGTTACTTCGGATTdTdT | AATCCGAAGTAACTTCCCTCGdGdG |
| siItgav-2 | GCCAGCCCATTGAGTTTGATTdTdT | AATCAAACTCAATGGGCTGGCdAdC |
| siFn1-1 | UGCCGUCUCUGUGCGCUAUUAdTdT | UGUAAUAGCGCACAGAGACGGdCdA |
| siFn1-2 | GCCAGCCCATTGAGTTTGATTdTdT | AATCAAACTCAATGGGCTGGCdAdC |
| siSpp1-1 | GGAUGAAUCUGACGAAUCUdTdT | AGAUUCGUCAGAUUCAUCCdGdA |
| siSpp1-2 | ACTCGGATGAATCTGACGAATdTdT | ATTCGTCAGATTCATCCGAGTdCdC |
| NC | UUCUCCGAACGUGUCACGUdTdT | ACGUGACACGUUCGGAGAAdTdT |
